# P-488. Epidemiology and Risk Factors of Pediatric Clostridioides difficile Infection Using a Nationwide Claims Database in Japan

**DOI:** 10.1093/ofid/ofaf695.703

**Published:** 2026-01-11

**Authors:** Daisuke Yamasaki, Yoshiki Kusama, Shiho Ito, Masaki Tanabe

**Affiliations:** Mie University Hospital, Tsu, Mie, Japan; University of Osaka Hospital, Suita, Osaka, Japan; Mie University, Tsu, Mie, Japan; Mie University Hospital, Tsu, Mie, Japan

## Abstract

**Background:**

*Clostridioides difficile* infection (CDI) is a leading cause of healthcare-associated diarrhea. In infants, symptomatic infection requiring treatment is rare due to immature intestinal receptors for the toxin. Thus, Guidelines recommend against CDI testing or treatment in children under two years of age unless alternative causes are excluded. Pediatric CDI often lacks traditional adult risk factors, and recent studies have reported increased community-onset CDI. However, nationwide data on pediatric CDI in Japan remain limited.

**Table 1:**
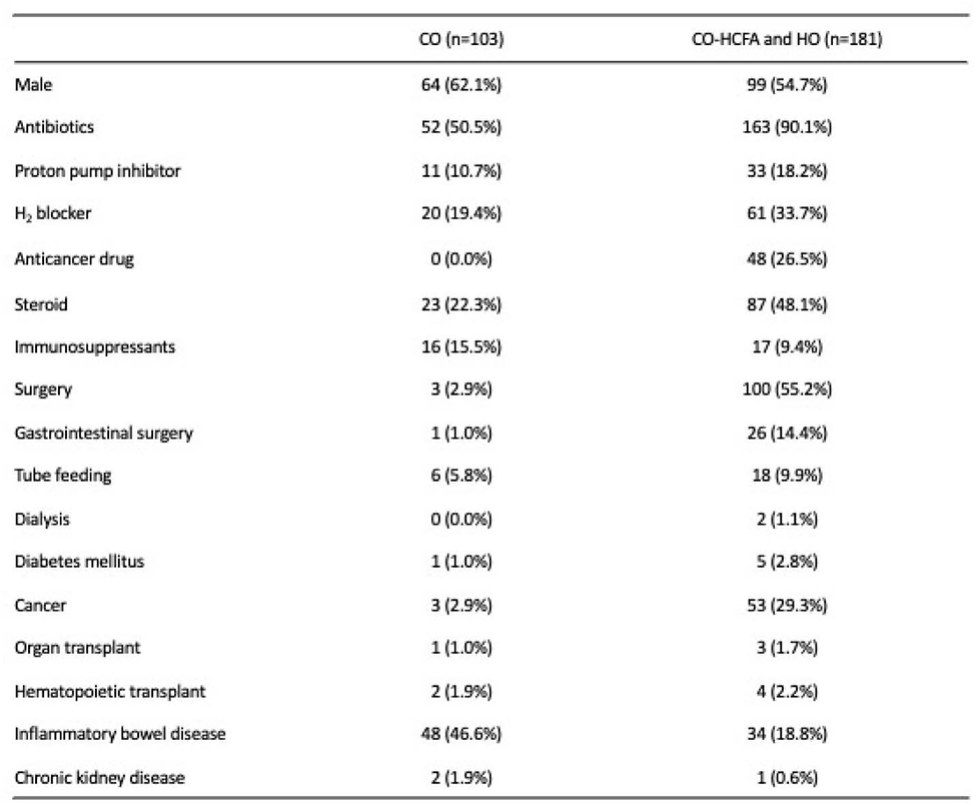
Comparison of risk factors by setting of onsets in pediatric CDI.

**Methods:**

We performed a retrospective observational study using JMDC (Japan Medical Data Center) claims data from 2013 to 2022. CDI was defined by the presence of both a claim for diagnostic testing (antigen or toxin B gene) and a prescription for anti-CDI medication within 7 days of testing. Episodes within 8 weeks of prior CDI were excluded. Patients were categorized by age (0–2, 3–5, 6–12, 13–17, 18–59, ≥60 years). CDI-onset setting was classified by U.S. CDC/NHSN infection surveillance protocol: hospital-onset (HO), healthcare facility–associated community-onset (HO-HCFA), and community-onset (CO). Risk factors within 30 days before onset were assessed across age groups and onset settings.

**Results:**

A total of 4,090 CDI cases met the definition, including 284 pediatric (0–17) cases. Although the incidence of CDI in children (0-17 years) was lower than in adults (18-59 years) and the elderly (60+ years), no significant difference was observed in all age groups of children, including 0-2 years. There was no significant increase in the incidence of CDI throughout the study period. Among pediatric cases, 75.7% had prior antibiotic use, 38.7% corticosteroids, and 28.9% IBD. Meanwhile, nearly half of pediatric CO-CDI cases lacked antibiotic exposure. Risk factor profiles varied by age and onset setting: enteral nutrition was most frequent in ages of 3–5, and inflammatory bowel disease in 13–17. The proportion of CO-CDI cases increased with age among children.

**Conclusion:**

This is the first large-scale study of pediatric CDI in Japan. Pediatric CDI occurred even in young children without antibiotic exposure, especially in the CO setting. These findings indicate a need to revise diagnostic and management strategies for pediatric CDI.

**Disclosures:**

All Authors: No reported disclosures

